# Supplementary material for: Cyy-287, a novel pyrimidine-2,4-diamine derivative, efficiently mitigates inflammatory responses, fibrosis, and lipid synthesis in obesity-induced cardiac and hepatic dysfunction
Source: PeerJ. 2024 Feb 29;12:e17009. doi: 10.7717/peerj.17009 (PMC10909366; doi:10.7717/peerj.17009)
Supplement: Supplemental Information 1 [file peerj-12-17009-s001.docx]

**Supplementary Figure 1**

**Real-time quantitative PCR**

Total RNA was extracted using TRIzol reagent (#10296028, Thermo Fisher Scientific, MA, USA). The first-strand cDNA template was synthesized using Hifair® Ⅲ 1st Strand cDNA Synthesis SuperMix (#11141ES60, yeasen, Shanghai, China). Real-time PCR was performed on a Bio-Rad CFX96 real-time system (BD, Franklin Lakes, USA) using Hieff qPCR SYBR Green Master Mix. GAPDH was employed as an internal control. PCR primers (Life Technologies, MA, USA) are listed in **supplementary table 1**.

**supFig.1 Cyy-287 attenuates the mRNA expression of SREBP-1c in HFD mice.** Quantified results were plotted. Data are presented as the mean ± SEM (n=3). *** *P* < 0.001 compared with the control group. ^##^ *P* < 0.01 compared with the HFD group.

**Supplementary Table 1**. Primers used for real-time qPCR assay.

| Gene | Species | ForwardPrimer | ReversedPrimer |
| --- | --- | --- | --- |
| SREBP-1c | Mouse | TGACCCGGCTATTCCGTGA | CTGGGCTGAGCAATACAGTTC |
| GAPDH | Mouse | AGGTCGGTGTGAACGGATTTG | TGTAGACCATGTAGTTGAGGTCA |
