# Supplementary material for: Cyy-287, a novel pyrimidine-2,4-diamine derivative, efficiently mitigates inflammatory responses, fibrosis, and lipid synthesis in obesity-induced cardiac and hepatic dysfunction
Source: PeerJ. 2024 Feb 29;12:e17009. doi: 10.7717/peerj.17009 (PMC10909366; doi:10.7717/peerj.17009)
Supplement: Supplemental Information 2 [file peerj-12-17009-s002.zip › Original data/Figure3. Heart tissue/Figure 3A. Heart tissue slices staining/heart tissue slices staining.pptx]

## Slide 1
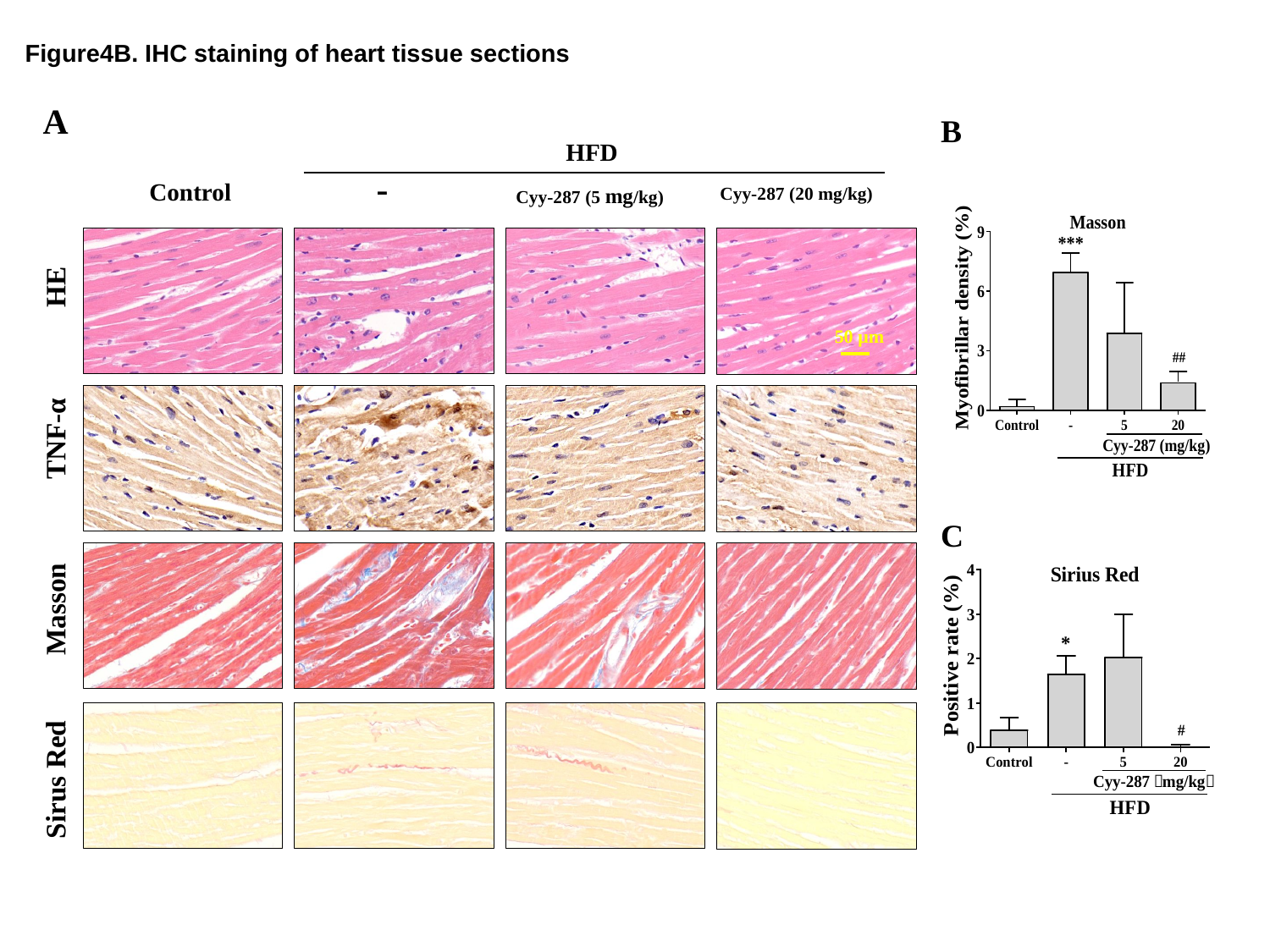

Figure4B. IHC staining of heart tissue sections
A
B
HFD
-
Cyy-287 (20 mg/kg)
Cyy-287 (5 mg/kg)
Control
HE
50 μm
TNF-α
C
Masson
Sirus Red
