# Supplementary material for: Cyy-287, a novel pyrimidine-2,4-diamine derivative, efficiently mitigates inflammatory responses, fibrosis, and lipid synthesis in obesity-induced cardiac and hepatic dysfunction
Source: PeerJ. 2024 Feb 29;12:e17009. doi: 10.7717/peerj.17009 (PMC10909366; doi:10.7717/peerj.17009)
Supplement: Supplemental Information 2 [file peerj-12-17009-s002.zip › Original data/Figure4. Liver tissue/Figure 4B. IHC staining/liver tissue slices staining.pptx]

## Slide 1
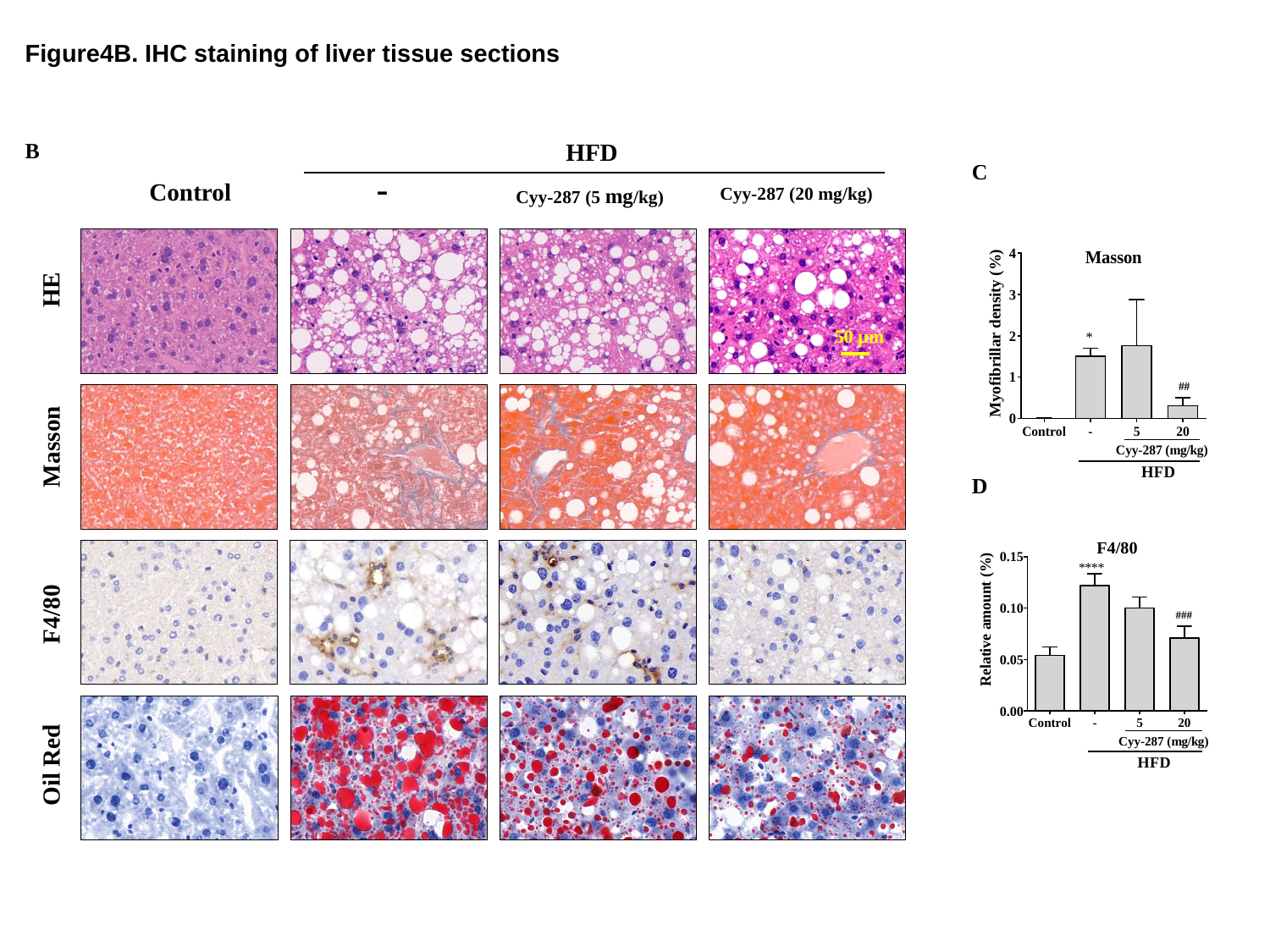

Figure4B. IHC staining of liver tissue sections
B
HFD
-
Cyy-287 (20 mg/kg)
Cyy-287 (5 mg/kg)
Control
C
HE
Masson
F4/80
Oil Red
50 μm
D
