# Supplementary material for: Cyy-287, a novel pyrimidine-2,4-diamine derivative, efficiently mitigates inflammatory responses, fibrosis, and lipid synthesis in obesity-induced cardiac and hepatic dysfunction
Source: PeerJ. 2024 Feb 29;12:e17009. doi: 10.7717/peerj.17009 (PMC10909366; doi:10.7717/peerj.17009)
Supplement: Supplemental Information 2 [file peerj-12-17009-s002.zip › Original data/Figure5. CYP450 detection/The original UV chromatograms of liver microsomes in Figure 5A.pptx]

## Slide 1
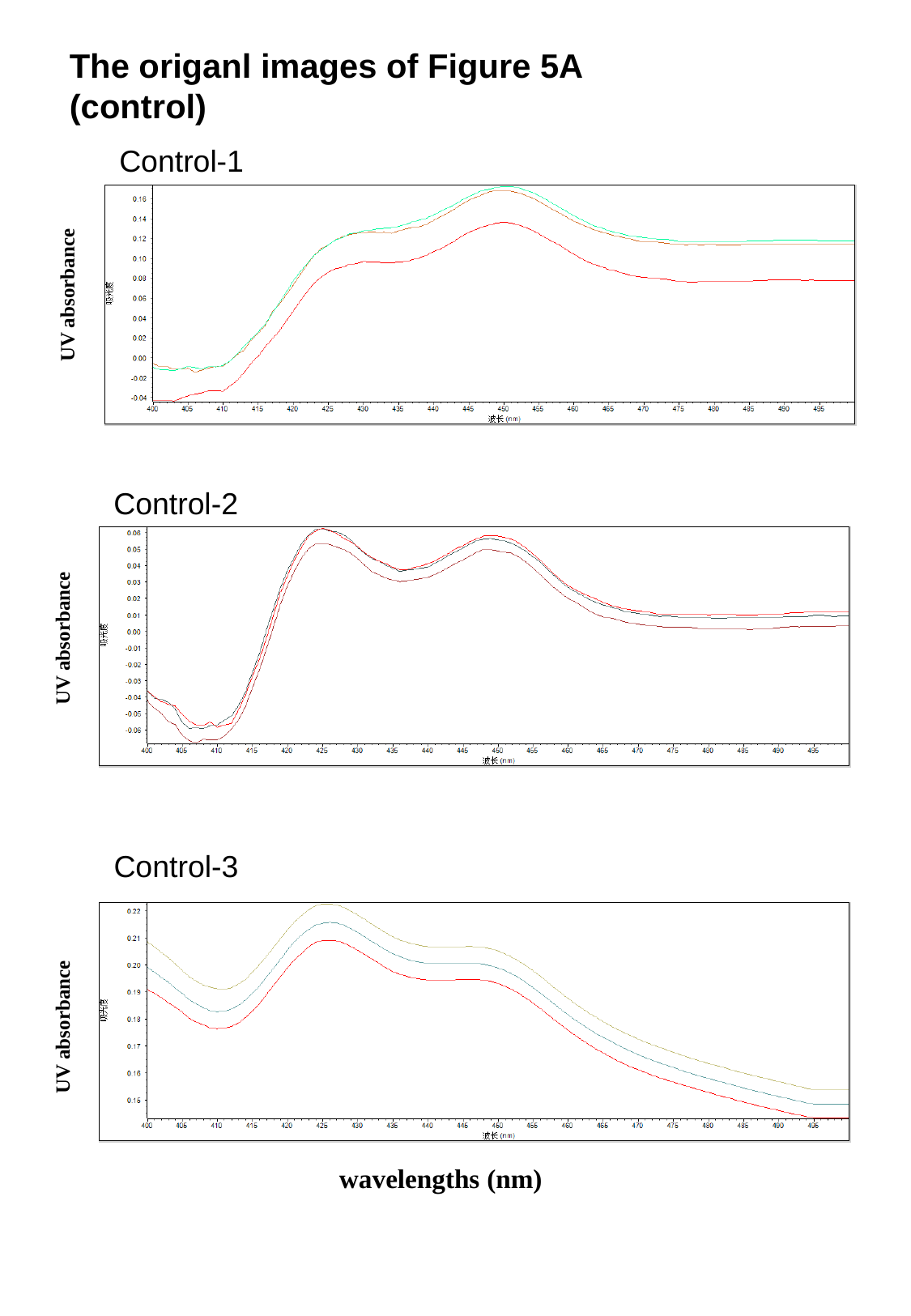

The origanl images of Figure 5A
(control)
Control-1
UV absorbance
Control-2
UV absorbance
Control-3
UV absorbance
wavelengths (nm)

## Slide 2
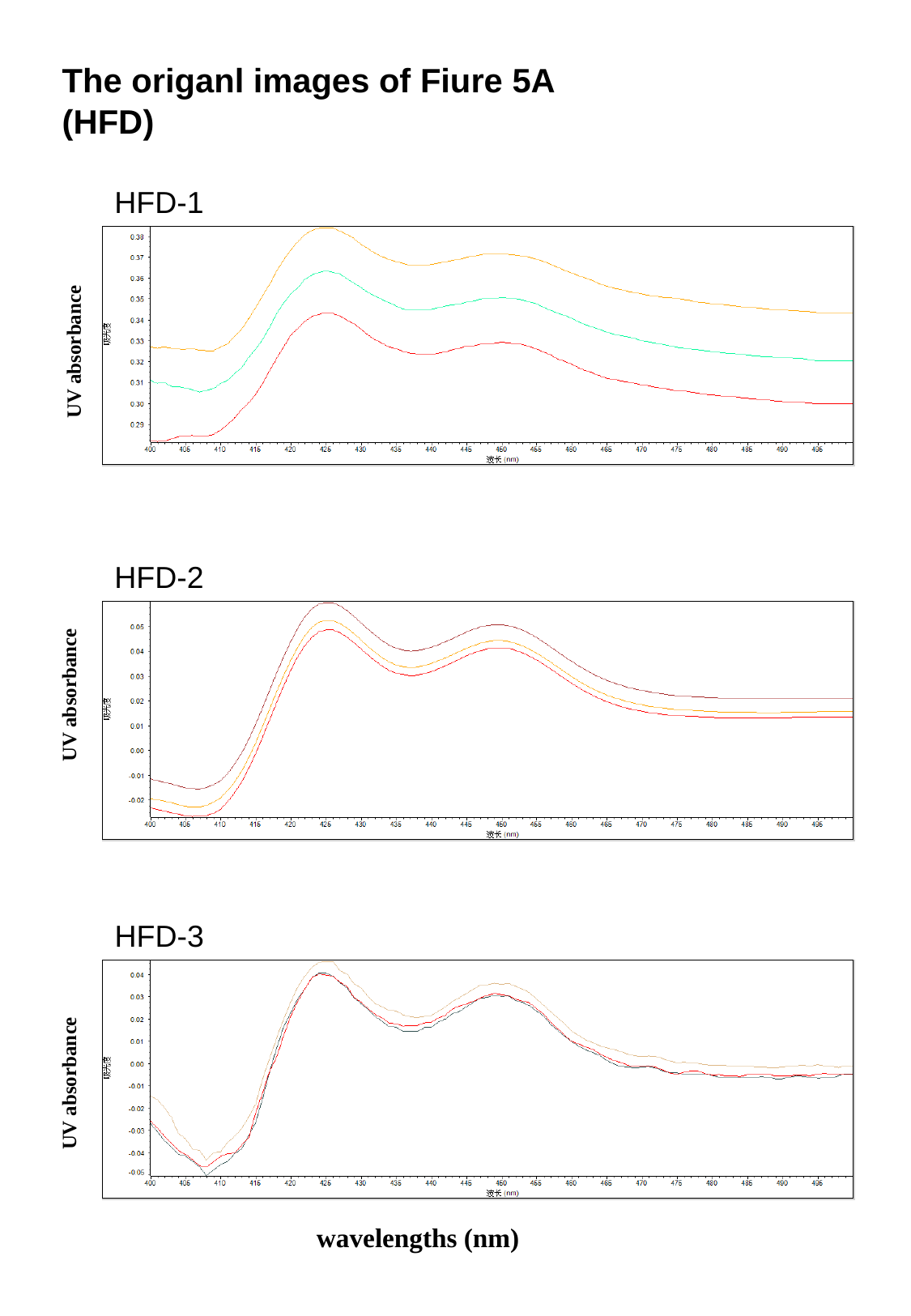

The origanl images of Fiure 5A
(HFD)
HFD-1
UV absorbance
HFD-2
UV absorbance
HFD-3
UV absorbance
wavelengths (nm)

## Slide 3
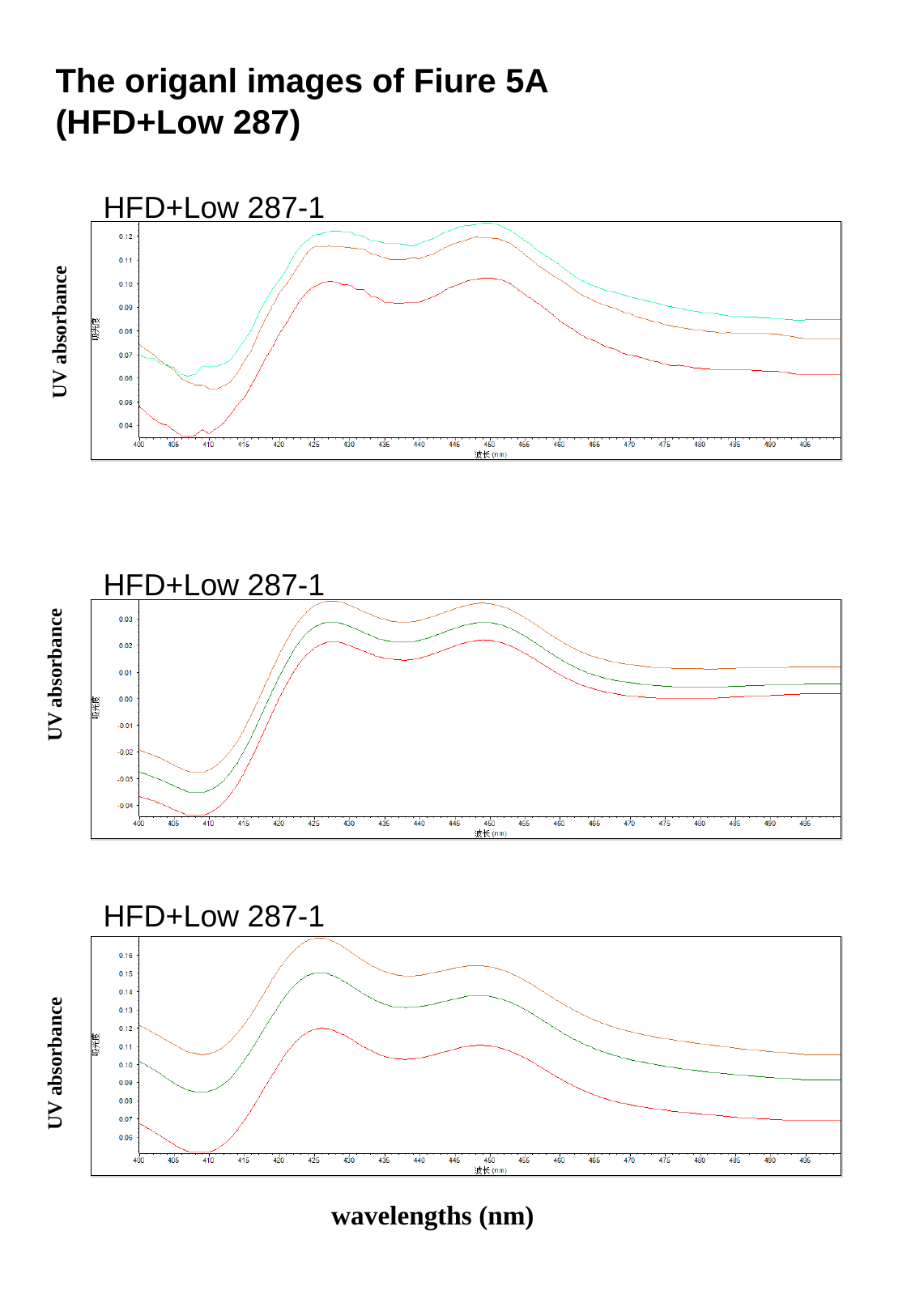

The origanl images of Fiure 5A
(HFD+Low 287)
HFD+Low 287-1
UV absorbance
HFD+Low 287-1
UV absorbance
HFD+Low 287-1
UV absorbance
wavelengths (nm)

## Slide 4
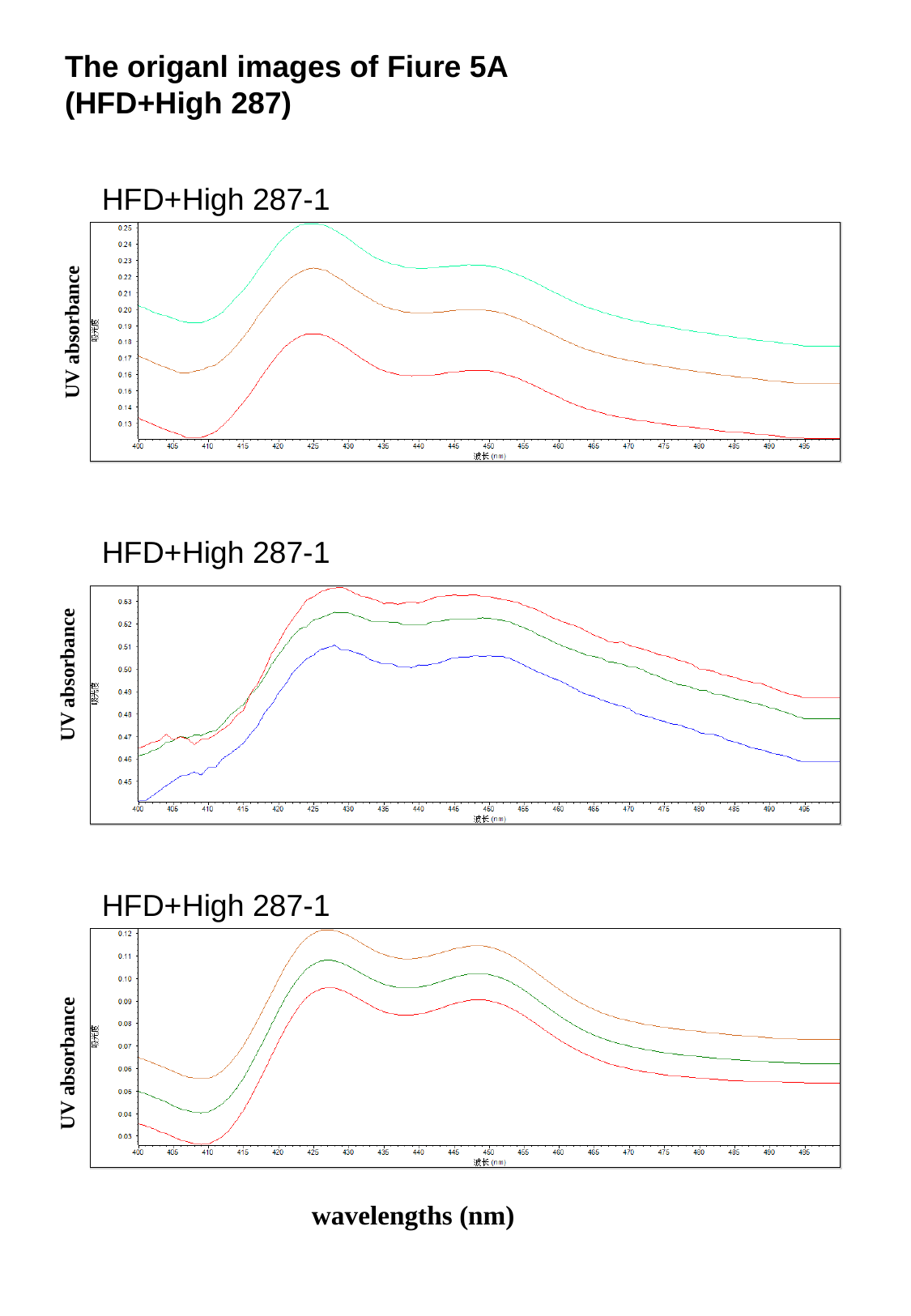

The origanl images of Fiure 5A
(HFD+High 287)
HFD+High 287-1
UV absorbance
HFD+High 287-1
UV absorbance
HFD+High 287-1
UV absorbance
wavelengths (nm)
