# Supplementary figures and images for: Cyy-287, a novel pyrimidine-2,4-diamine derivative, efficiently mitigates inflammatory responses, fibrosis, and lipid synthesis in obesity-induced cardiac and hepatic dysfunction
Source: PeerJ. 2024 Feb 29;12:e17009. doi: 10.7717/peerj.17009 (PMC10909366; doi:10.7717/peerj.17009)

## Slide 1
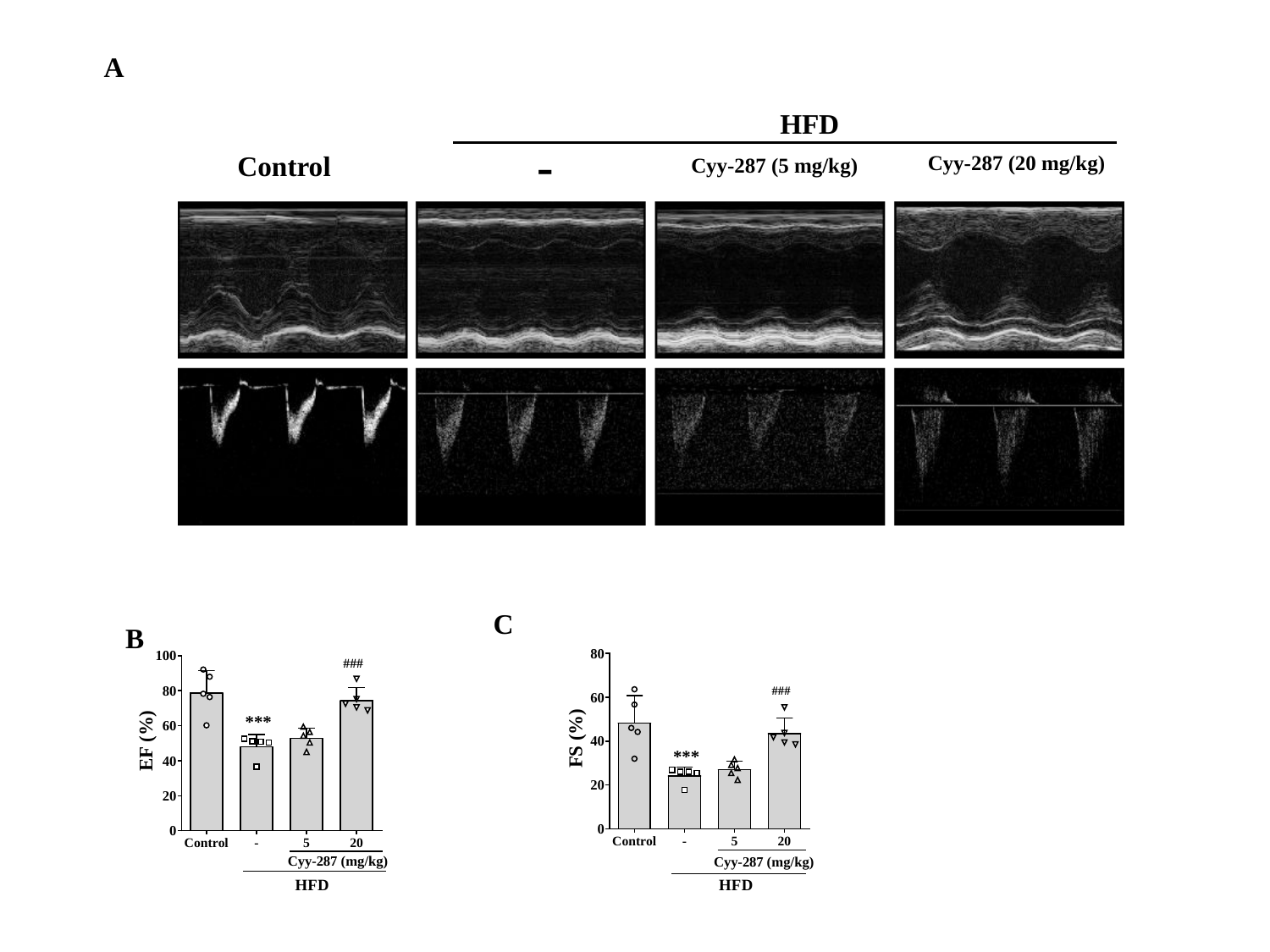

A
HFD
-
Cyy-287 (20 mg/kg)
Cyy-287 (5 mg/kg)
Control
C
B

Supplement: Supplemental Information 2 [file peerj-12-17009-s002.zip › Original data/Figure 2. ECHO image, serum ALT and AST/Figure 2A. echocardiography image/echocardiography image.pptx]

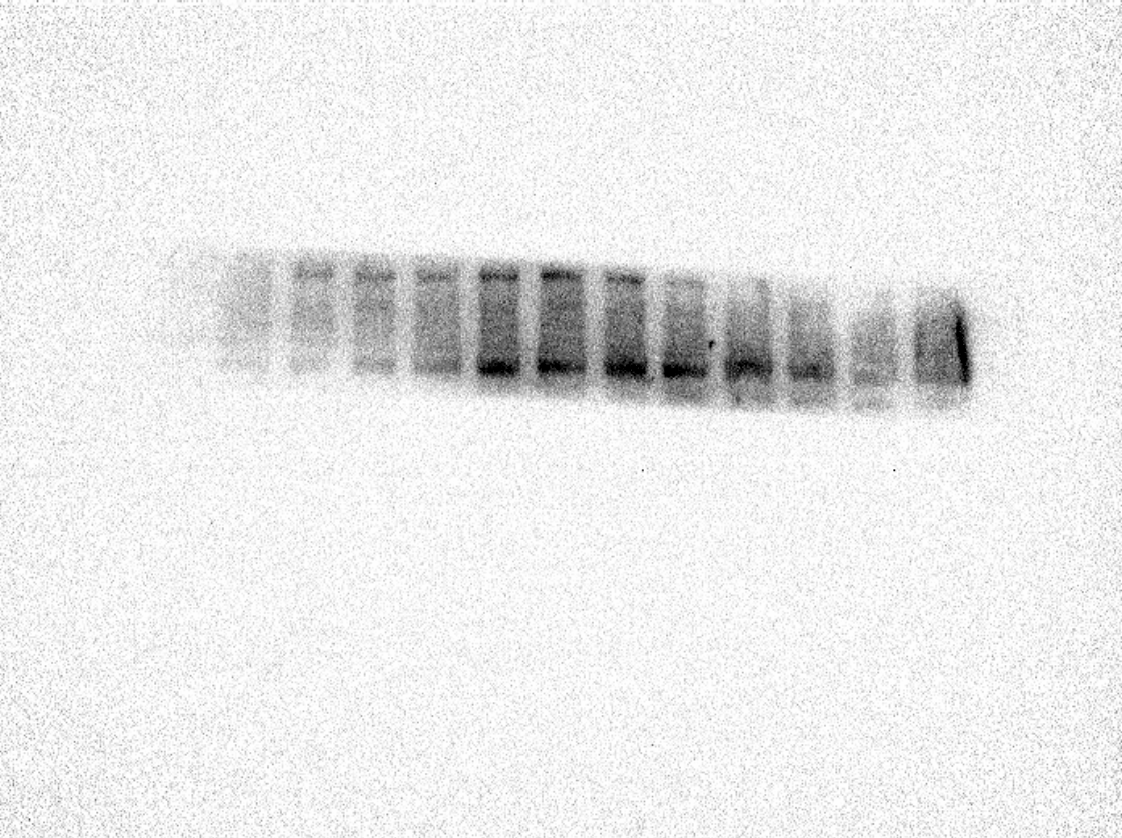

Supplement: Supplemental Information 2 [file peerj-12-17009-s002.zip › Original data/Figure3. Heart tissue/Figure 3D. protein bands/1 Collagen1 and P-AMPK/heart COL-1.tif]

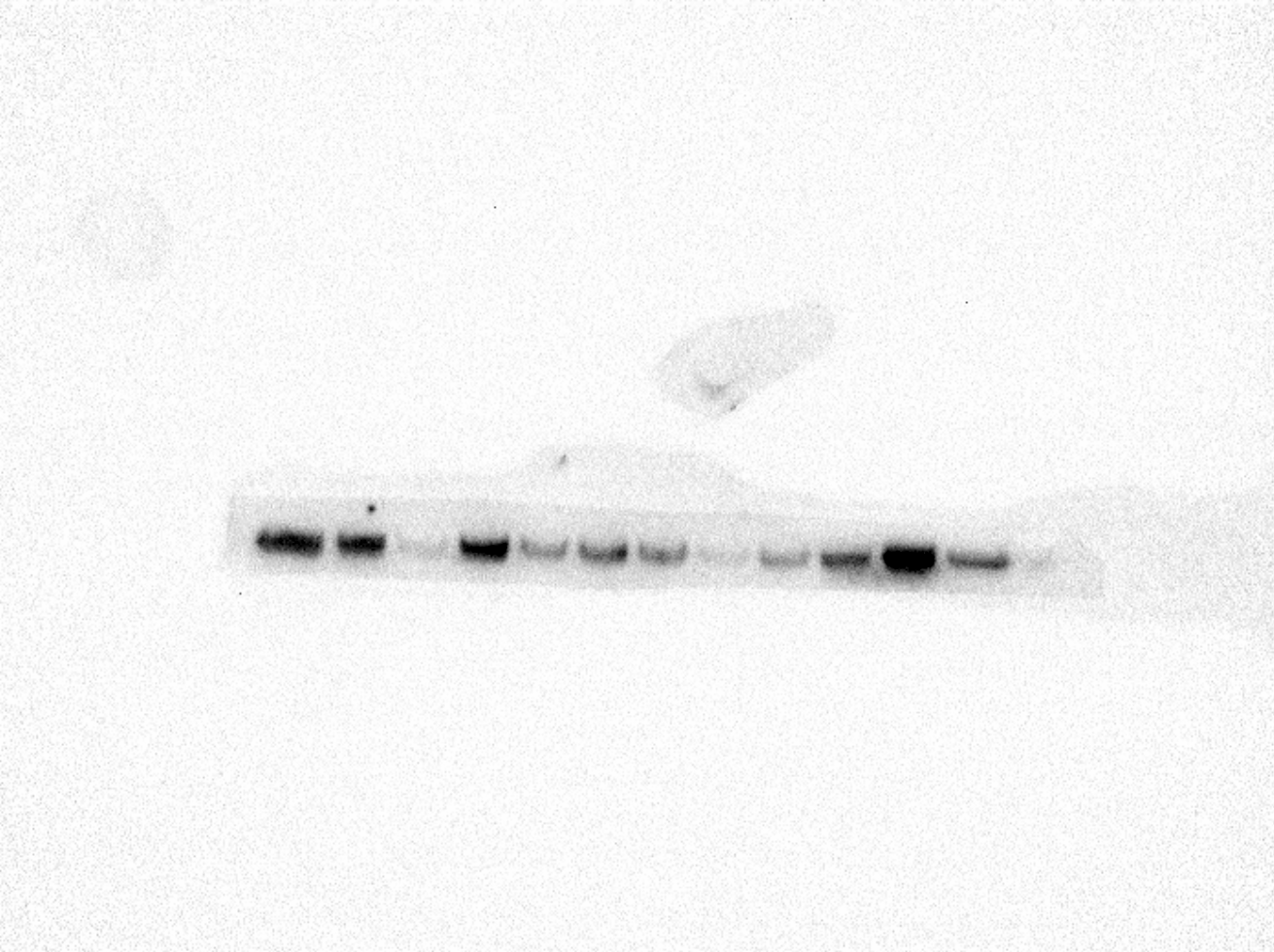

Supplement: Supplemental Information 2 [file peerj-12-17009-s002.zip › Original data/Figure3. Heart tissue/Figure 3D. protein bands/1 Collagen1 and P-AMPK/heart P-AMPK.tif]

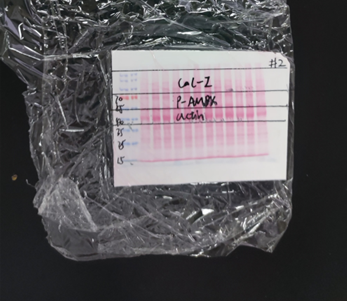

Supplement: Supplemental Information 2 [file peerj-12-17009-s002.zip › Original data/Figure3. Heart tissue/Figure 3D. protein bands/1 Collagen1 and P-AMPK/membrane No.1.tif]

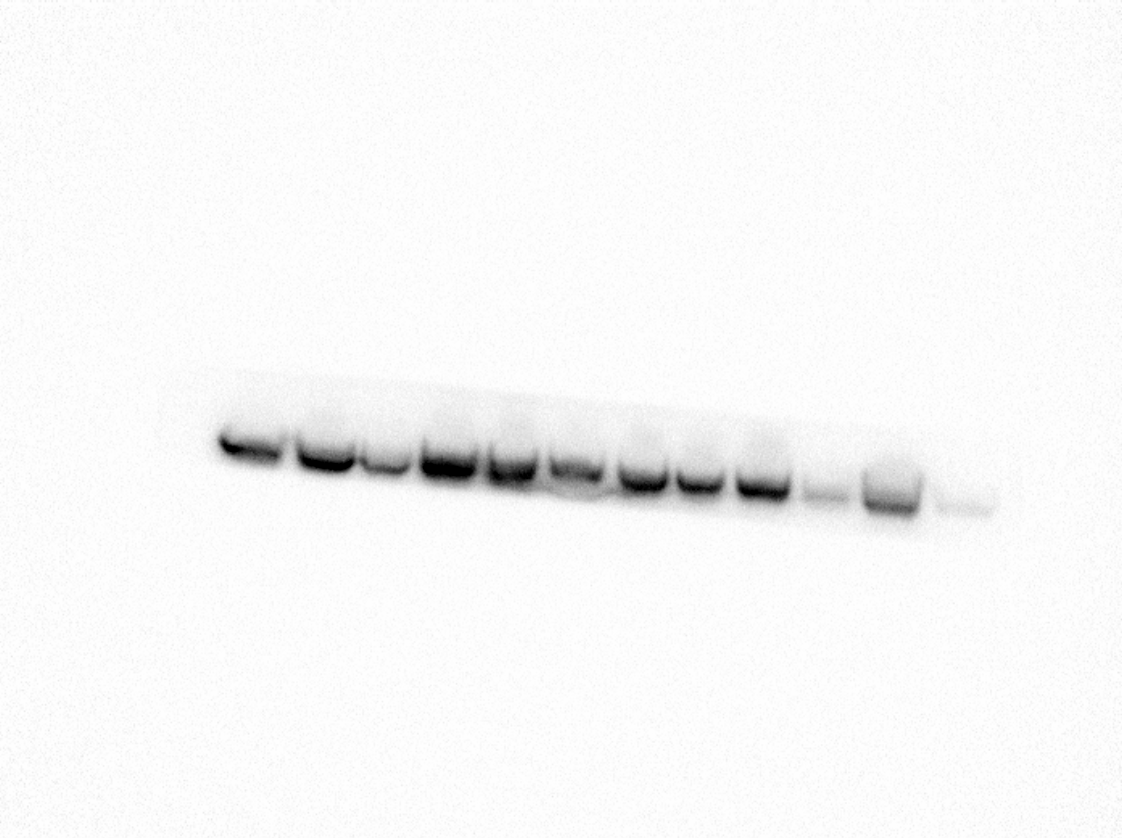

Supplement: Supplemental Information 2 [file peerj-12-17009-s002.zip › Original data/Figure3. Heart tissue/Figure 3D. protein bands/2 P-p65, P-p38 and tublin/heart P-P38.tif]

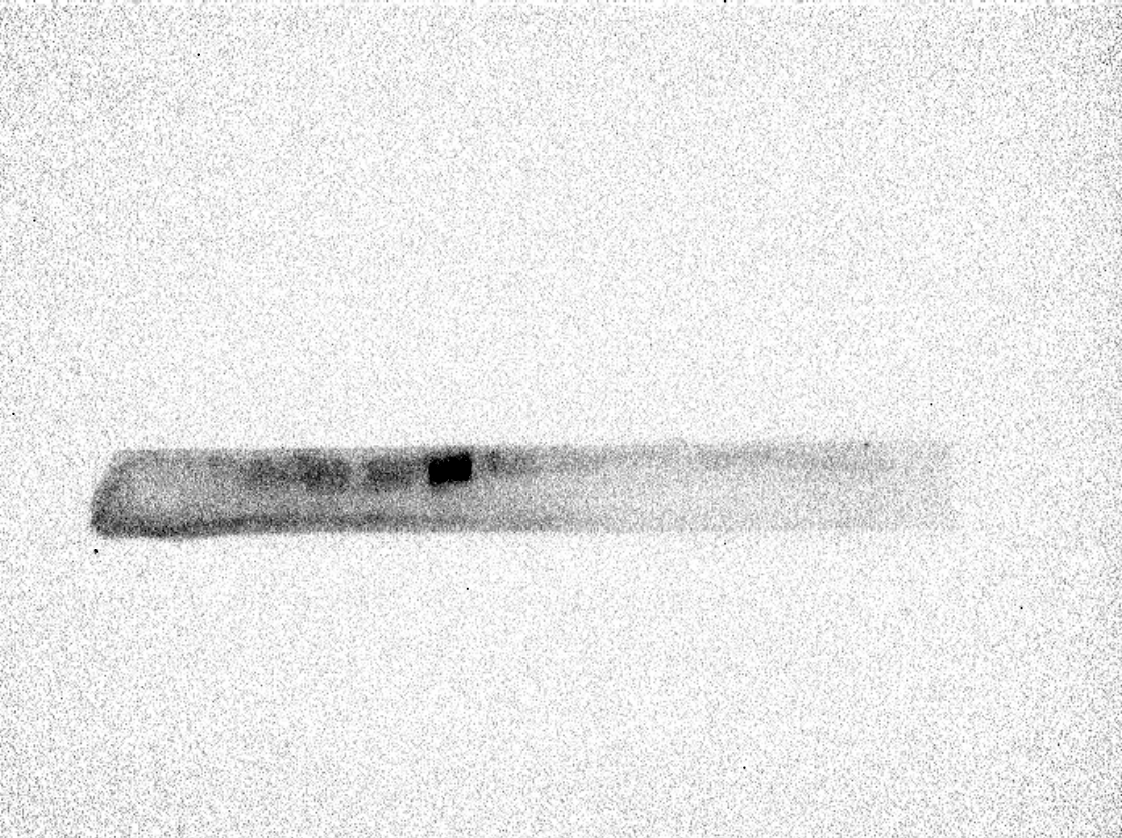

Supplement: Supplemental Information 2 [file peerj-12-17009-s002.zip › Original data/Figure3. Heart tissue/Figure 3D. protein bands/2 P-p65, P-p38 and tublin/heart P-P65.tif]

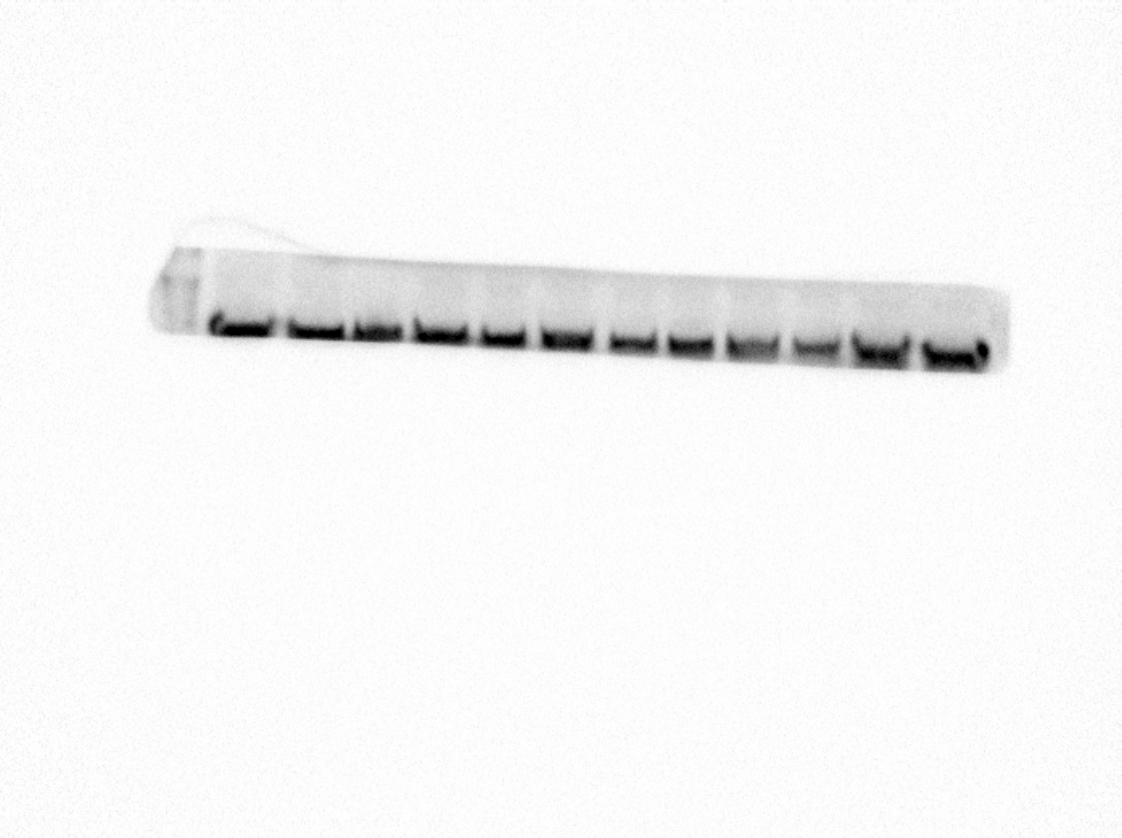

Supplement: Supplemental Information 2 [file peerj-12-17009-s002.zip › Original data/Figure3. Heart tissue/Figure 3D. protein bands/2 P-p65, P-p38 and tublin/heart tubulin .tif]

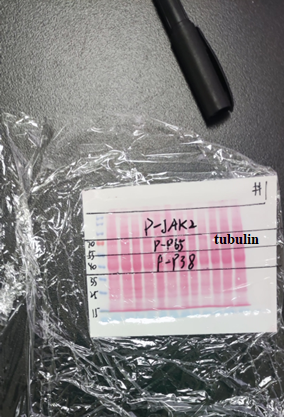

Supplement: Supplemental Information 2 [file peerj-12-17009-s002.zip › Original data/Figure3. Heart tissue/Figure 3D. protein bands/2 P-p65, P-p38 and tublin/membrane No.2.tif]

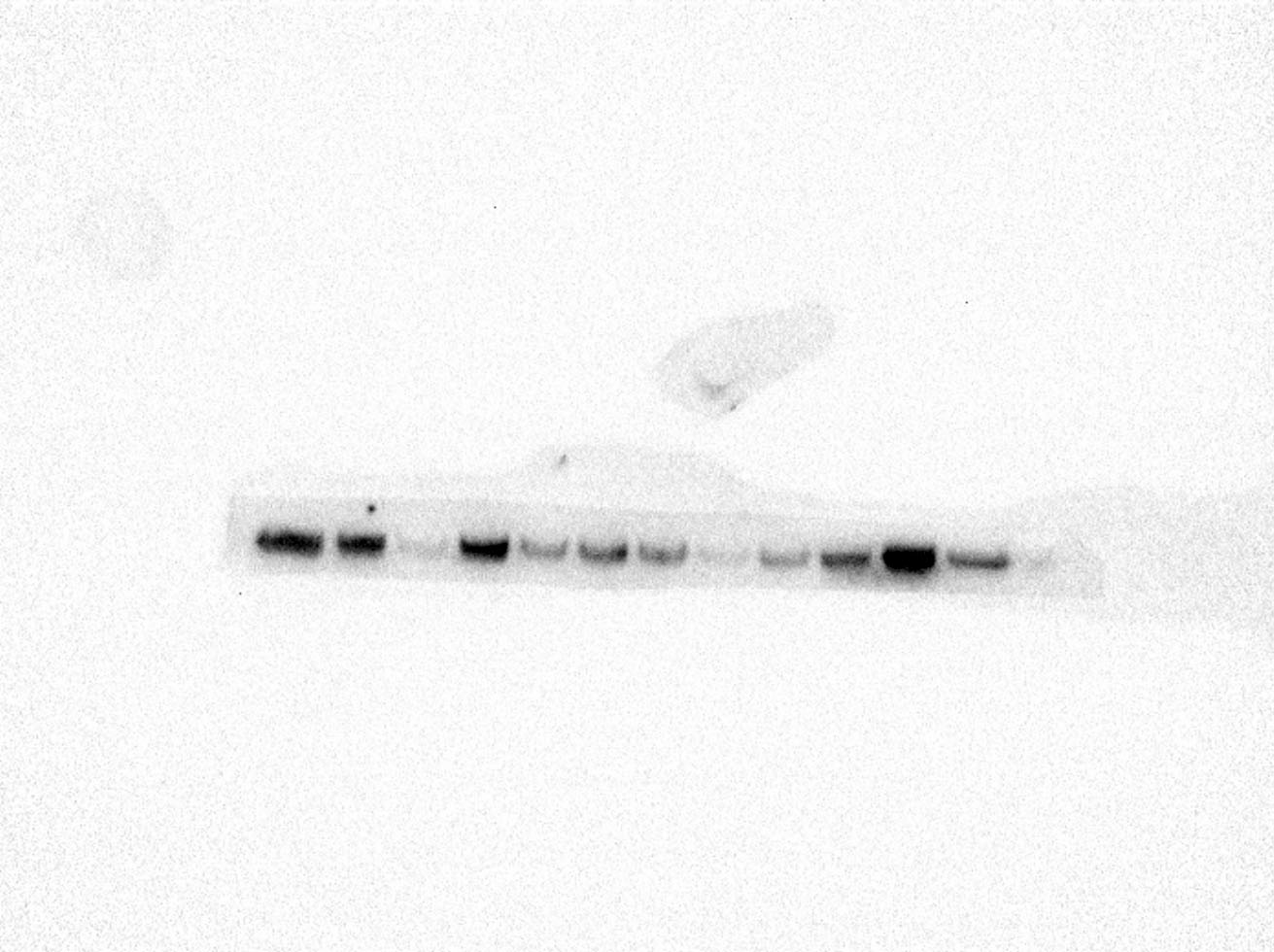

Supplement: Supplemental Information 2 [file peerj-12-17009-s002.zip › Original data/Figure3. Heart tissue/Figure 3D. protein bands/3 p38/heart P-AMPK.jpg]

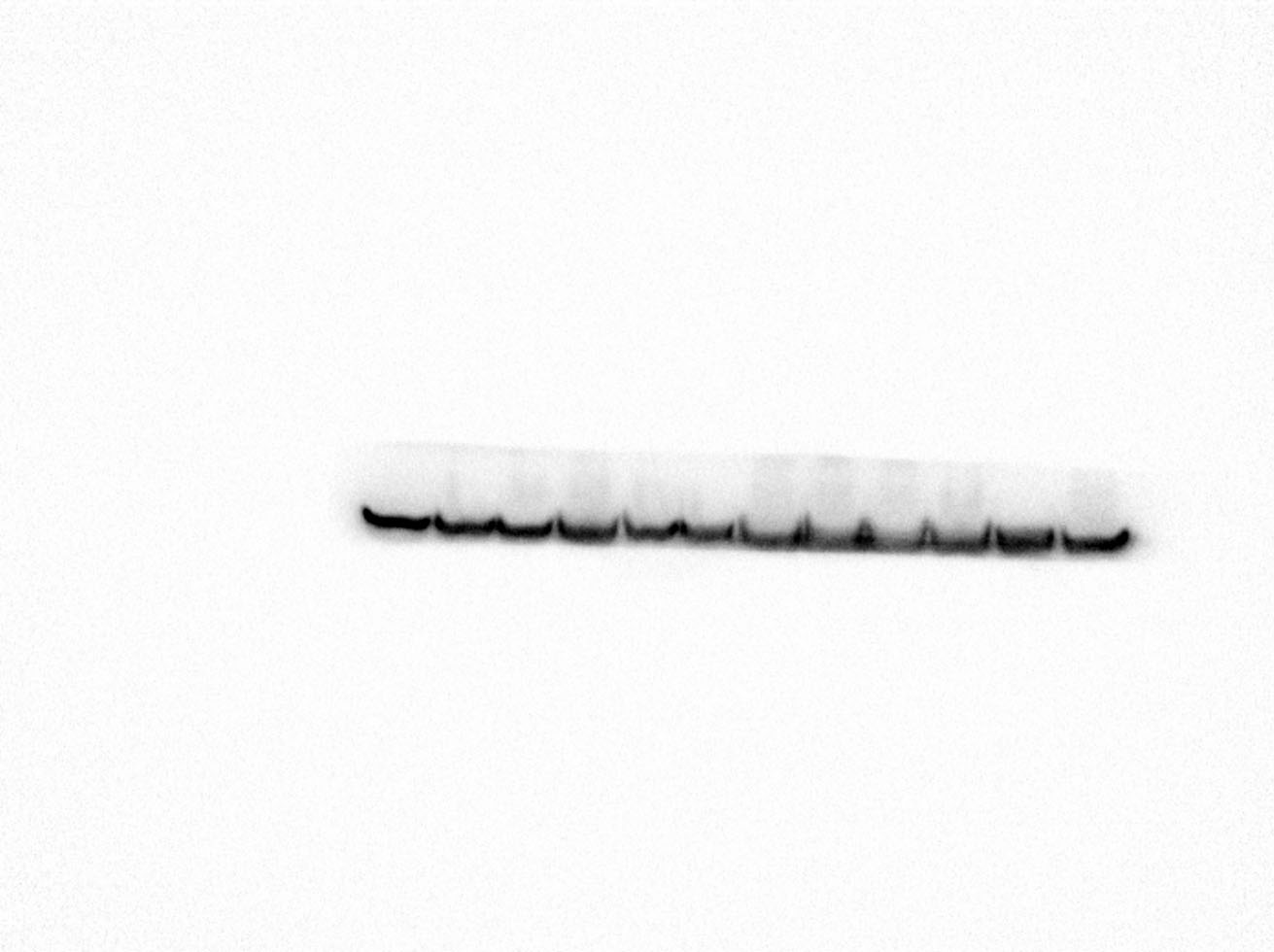

Supplement: Supplemental Information 2 [file peerj-12-17009-s002.zip › Original data/Figure3. Heart tissue/Figure 3D. protein bands/3 p38/heart P38.jpg]

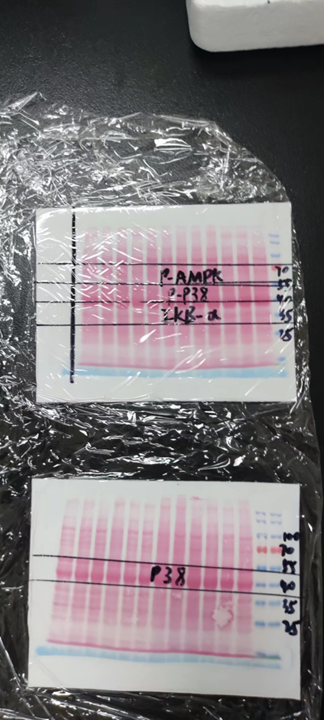

Supplement: Supplemental Information 2 [file peerj-12-17009-s002.zip › Original data/Figure3. Heart tissue/Figure 3D. protein bands/3 p38/membrane No.3.tif]

## Slide 1
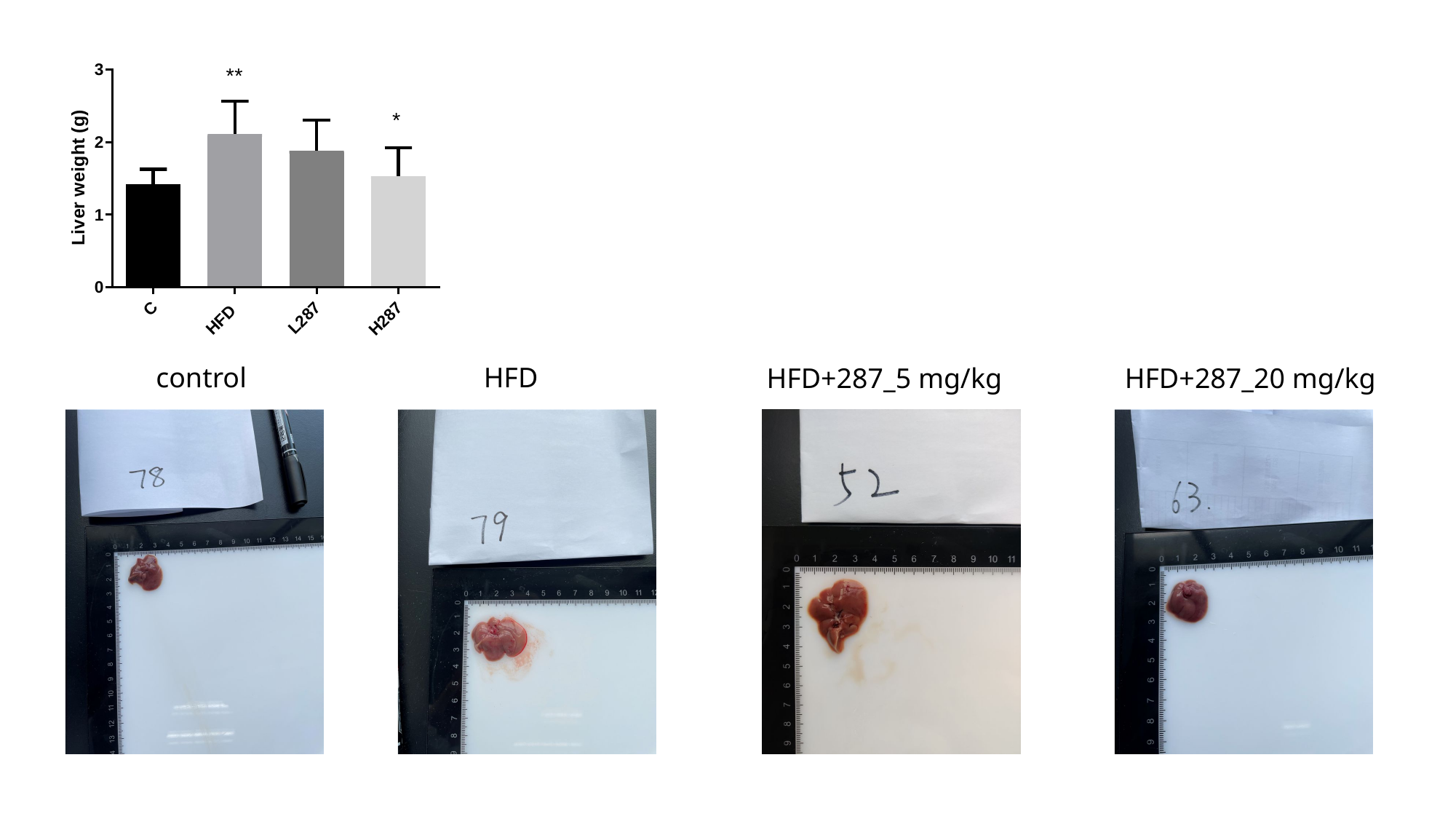

control
HFD
HFD+287_5 mg/kg
HFD+287_20 mg/kg

Supplement: Supplemental Information 2 [file peerj-12-17009-s002.zip › Original data/Figure4. Liver tissue/Figure 4A. The gross images of livers/the gross images of livers.pptx]

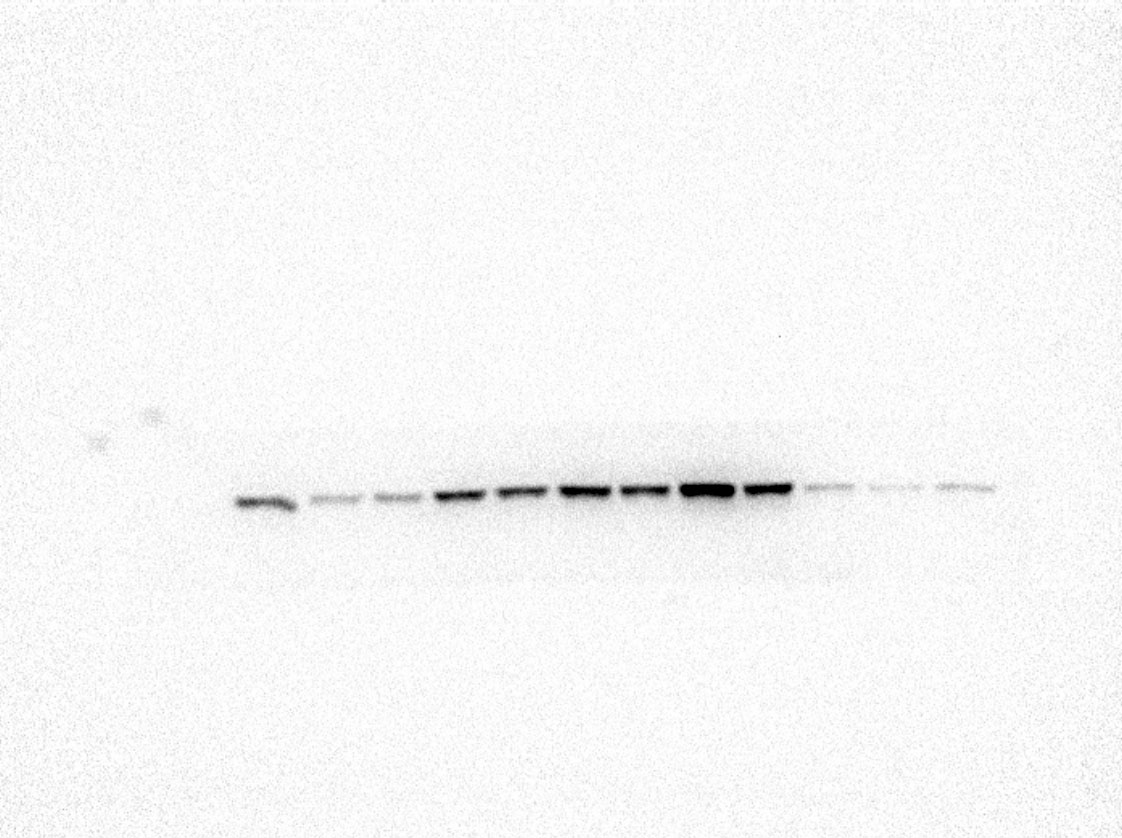

Supplement: Supplemental Information 2 [file peerj-12-17009-s002.zip › Original data/Figure4. Liver tissue/Figure 4E. protein bands/1 P-p38/liver P-P38.jpg]

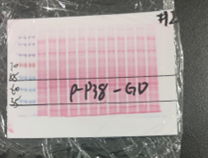

Supplement: Supplemental Information 2 [file peerj-12-17009-s002.zip › Original data/Figure4. Liver tissue/Figure 4E. protein bands/1 P-p38/membrane No.1.tif]

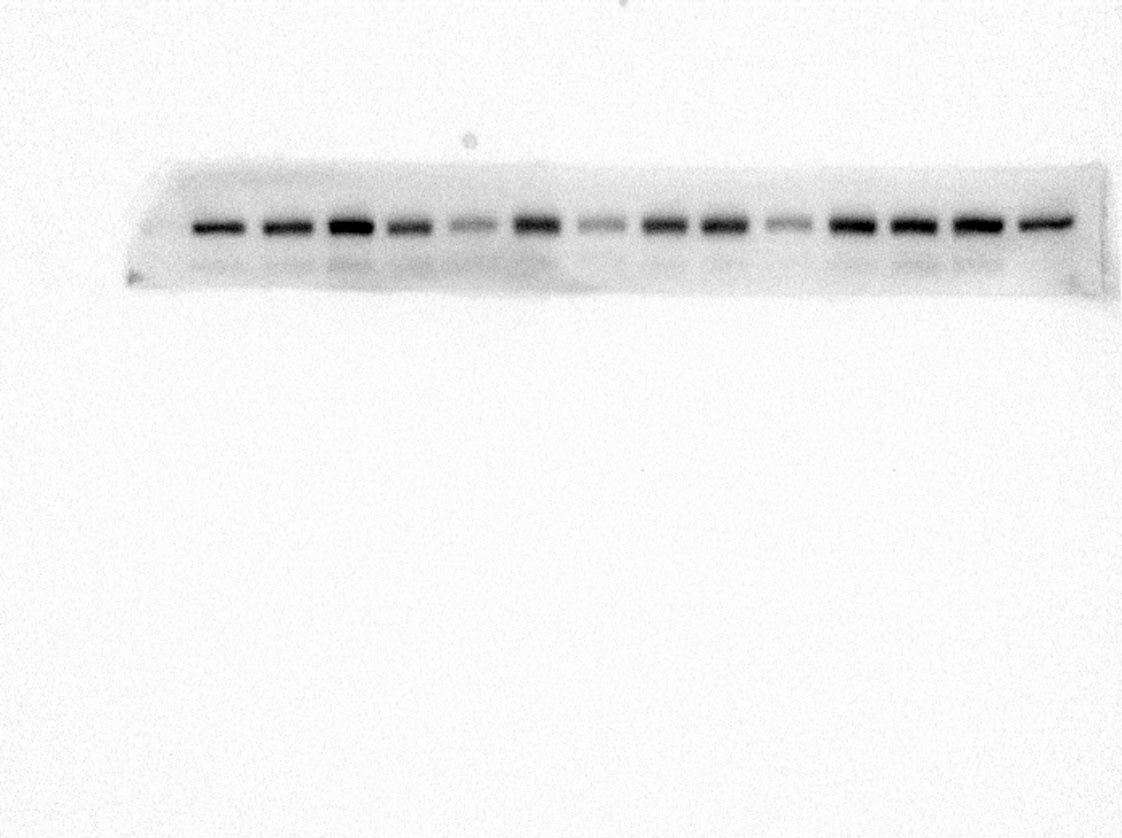

Supplement: Supplemental Information 2 [file peerj-12-17009-s002.zip › Original data/Figure4. Liver tissue/Figure 4E. protein bands/2 p38, P-AMPK and P-p65/liver P-AMPK.jpg]

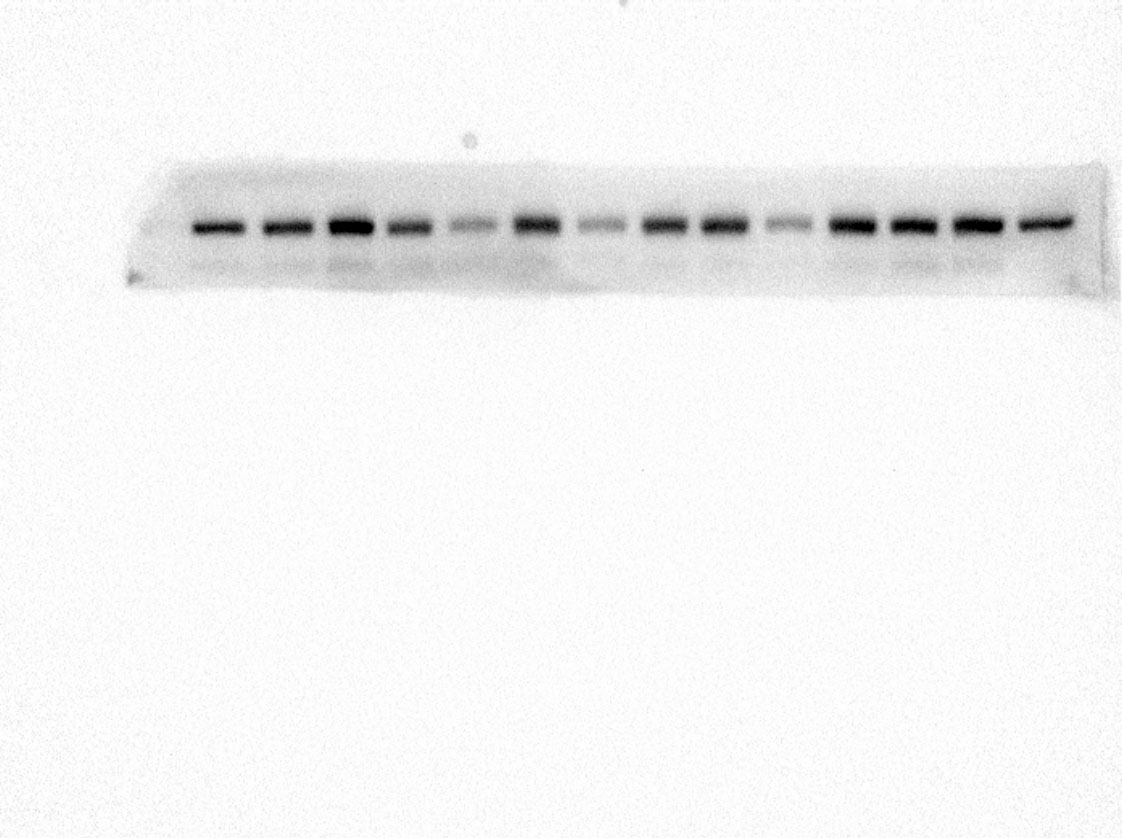

Supplement: Supplemental Information 2 [file peerj-12-17009-s002.zip › Original data/Figure4. Liver tissue/Figure 4E. protein bands/2 p38, P-AMPK and P-p65/liver P-P65.jpg]

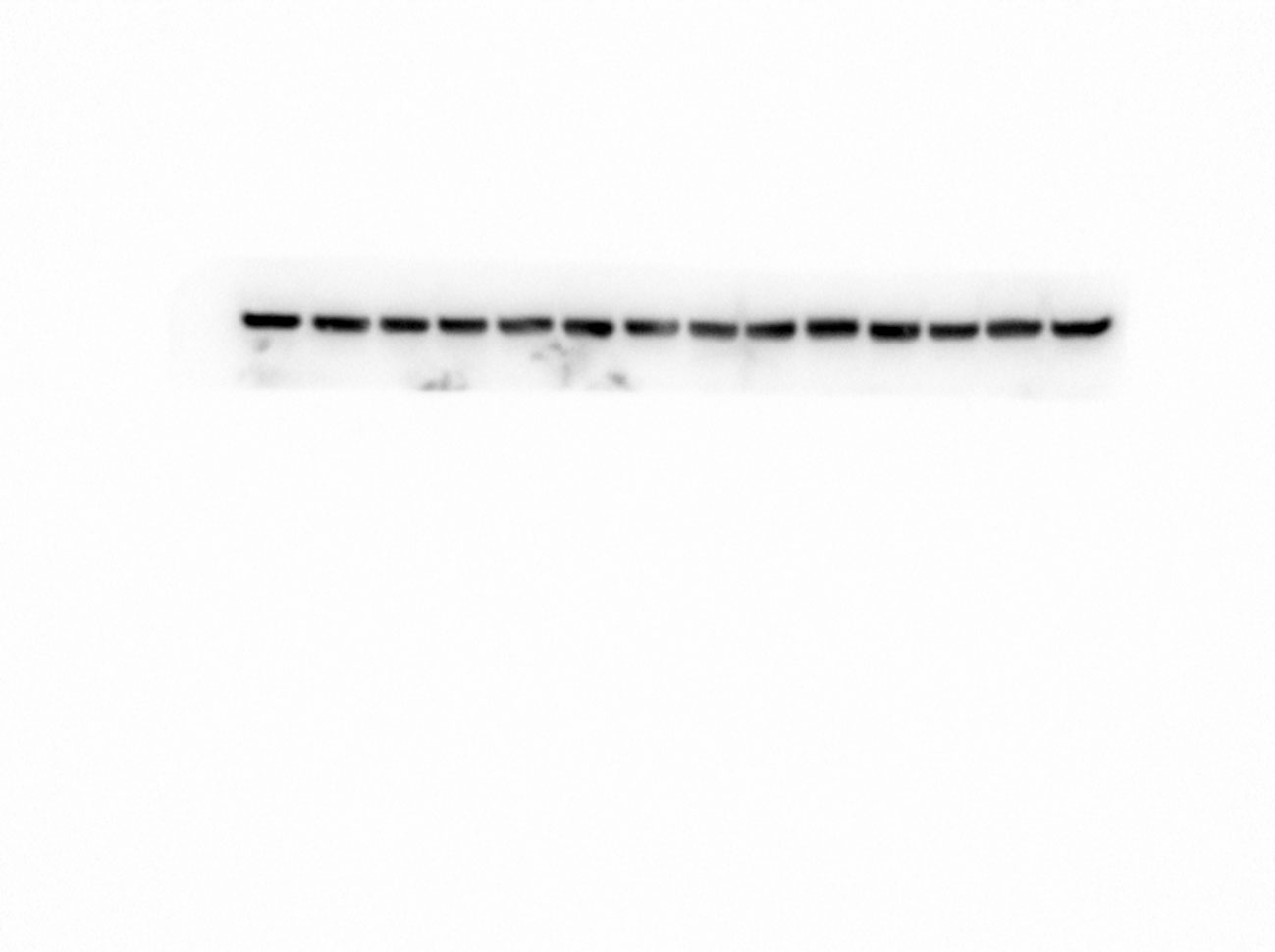

Supplement: Supplemental Information 2 [file peerj-12-17009-s002.zip › Original data/Figure4. Liver tissue/Figure 4E. protein bands/2 p38, P-AMPK and P-p65/liver P38.jpg]

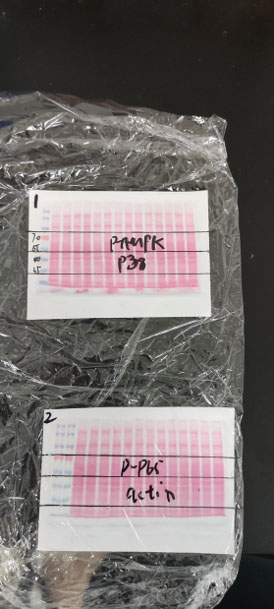

Supplement: Supplemental Information 2 [file peerj-12-17009-s002.zip › Original data/Figure4. Liver tissue/Figure 4E. protein bands/2 p38, P-AMPK and P-p65/membrane No2 and 3.jpg]

## Slide 1
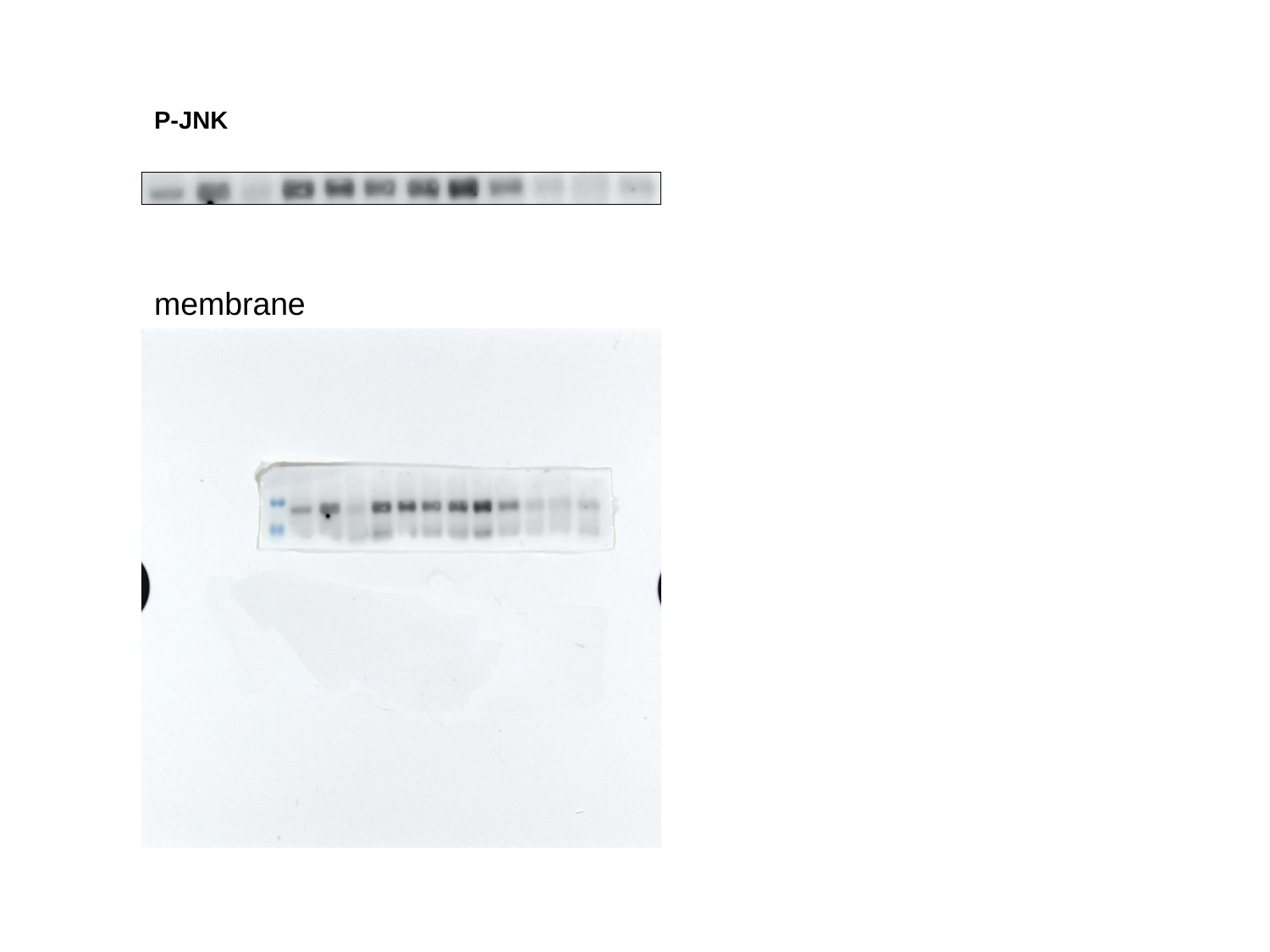

P-JNK
membrane

Supplement: Supplemental Information 2 [file peerj-12-17009-s002.zip › Original data/Figure4. Liver tissue/Figure 4E. protein bands/3 P-jNK/P-JNK protein band.pptx]

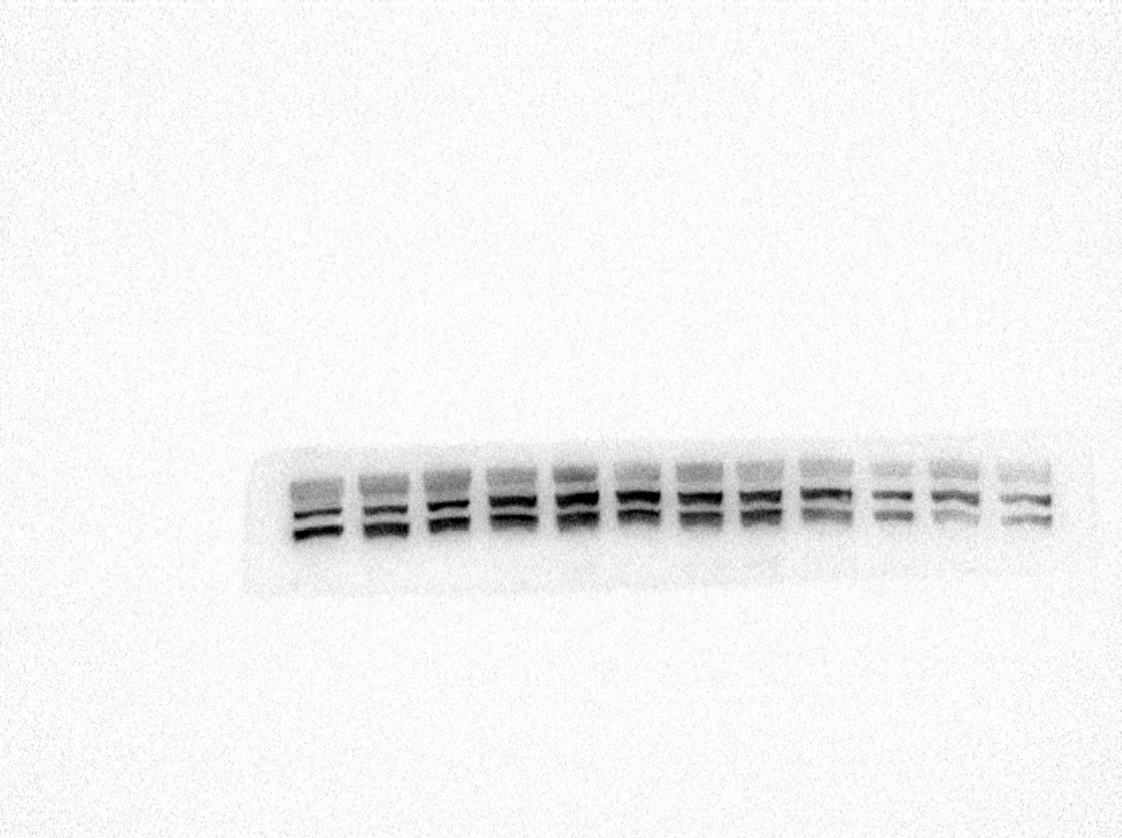

Supplement: Supplemental Information 2 [file peerj-12-17009-s002.zip › Original data/Figure4. Liver tissue/Figure 4E. protein bands/4 Collagen1 and tubulin/liver COL-1.tif]

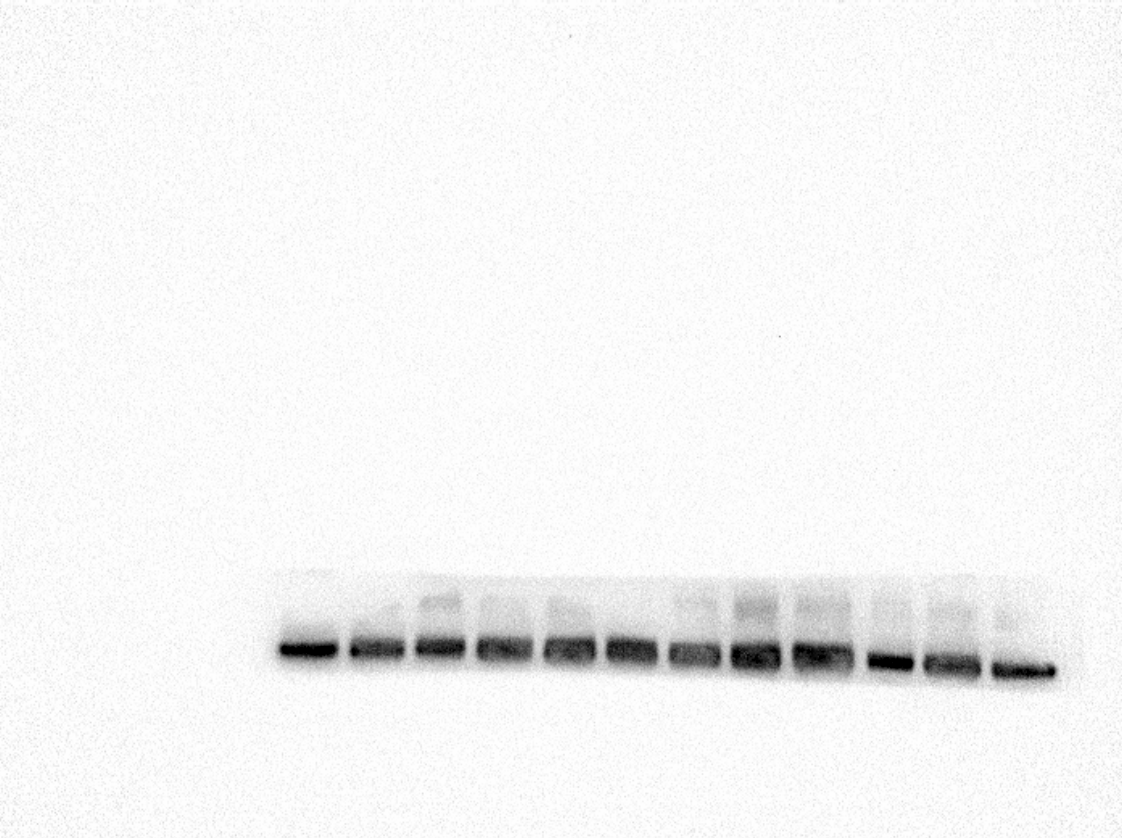

Supplement: Supplemental Information 2 [file peerj-12-17009-s002.zip › Original data/Figure4. Liver tissue/Figure 4E. protein bands/4 Collagen1 and tubulin/liver tubulin.tif]

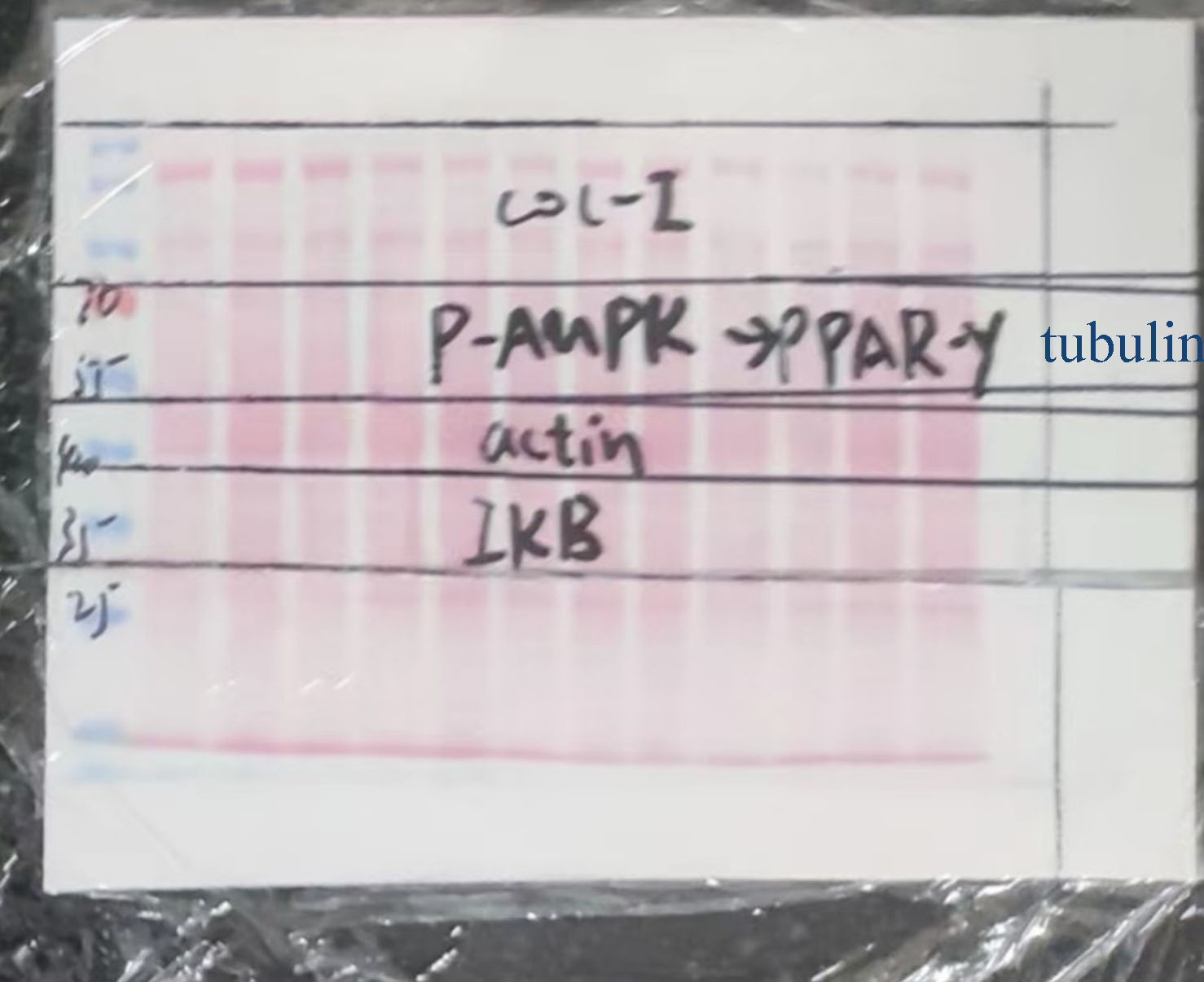

Supplement: Supplemental Information 2 [file peerj-12-17009-s002.zip › Original data/Figure4. Liver tissue/Figure 4E. protein bands/4 Collagen1 and tubulin/membrane No5.jpg]

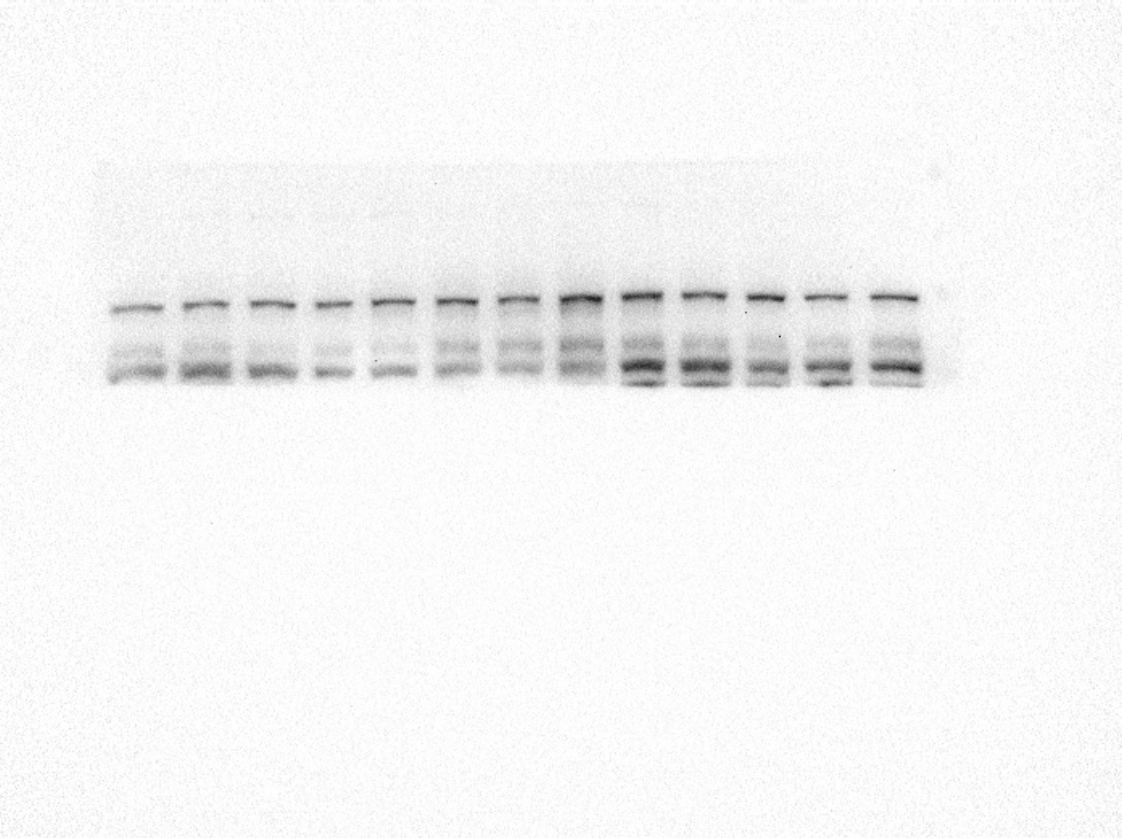

Supplement: Supplemental Information 2 [file peerj-12-17009-s002.zip › Original data/Figure4. Liver tissue/Figure 4E. protein bands/5 SREBP1c/liver SREBP1C.tif]

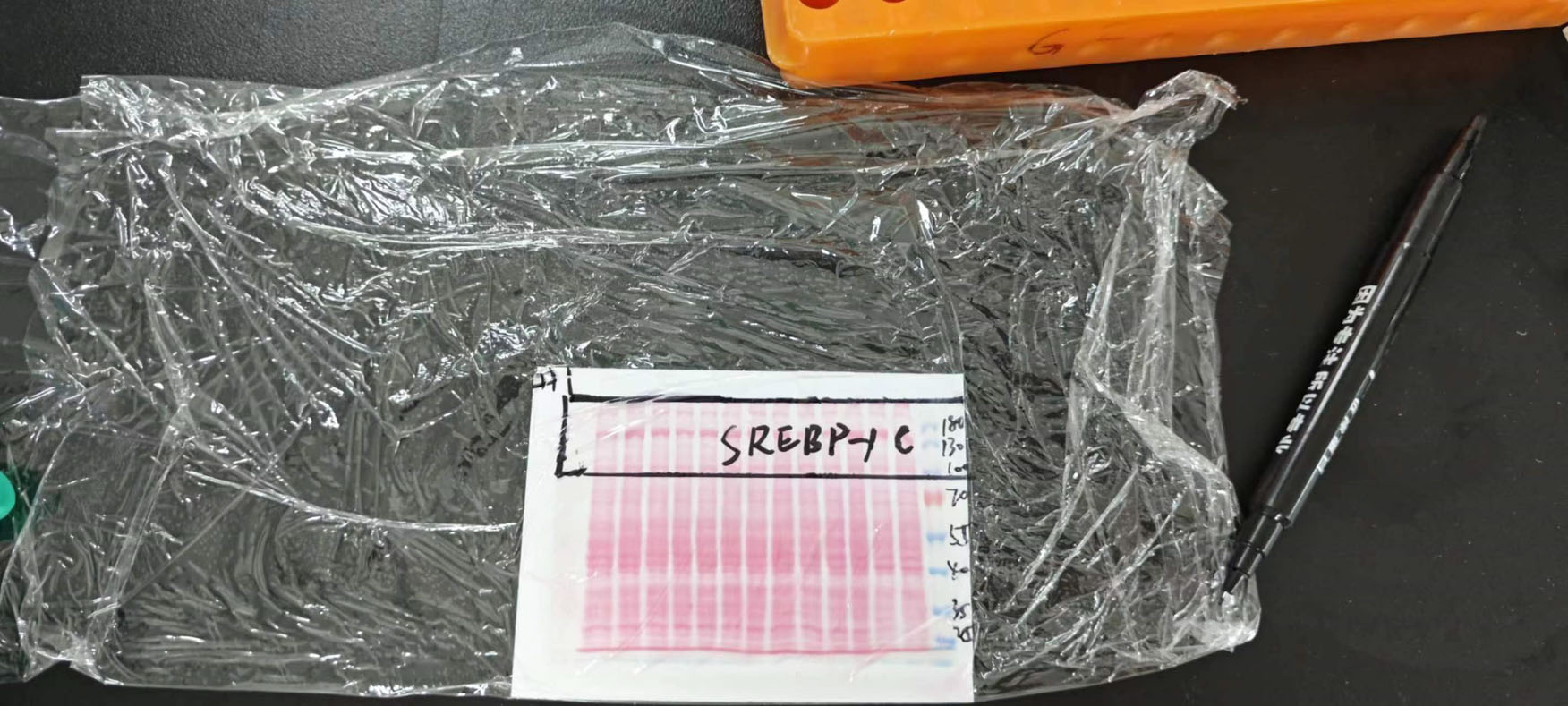

Supplement: Supplemental Information 2 [file peerj-12-17009-s002.zip › Original data/Figure4. Liver tissue/Figure 4E. protein bands/5 SREBP1c/membrane No5.jpg]
